# Tissue-Specific Accumulation and Isomerization of Valuable Phenylethanoid Glycosides from *Plantago* and *Forsythia* Plants

**DOI:** 10.3390/ijms22083880

**Published:** 2021-04-09

**Authors:** Moritz Zürn, Gergő Tóth, Tim Ausbüttel, Zoltán Mucsi, Kata Horváti, Szilvia Bősze, Magdolna Sütöri-Diószegi, Bernadett Pályi, Zoltán Kis, Béla Noszál, Imre Boldizsár

**Affiliations:** 1 Department of Plant Anatomy, Institute of Biology, Eötvös Loránd University, Pázmány Péter Sétány 1/C, 1117 Budapest, Hungary; moritz.zuern@gmail.com (M.Z.); tim.ausbuettel@gmail.com (T.A.); 2 Department of Pharmaceutical Chemistry, Semmelweis University, Hőgyes Endre u. 9, 1092 Budapest, Hungary; toth.gergo@pharma.semmelweis-univ.hu (G.T.); noszal.bela@pharma.semmelweis-univ.hu (B.N.); 3 Department of Pharmacognosy, Semmelweis University, Üllői út 26, 1085 Budapest, Hungary; 4 Femtonics Ltd., Tűzoltó u. 59, 1094 Budapest, Hungary; zmucsi@femtonics.eu; 5 Research Group of Peptide Chemistry, Eötvös Loránd University, Eötvös Loránd Research Network (ELKH), Pázmány Péter Sétány 1/A, 1117 Budapest, Hungary; khorvati@elte.hu (K.H.); szilvia.bosze@ttk.elte.hu (S.B.); 6 Department of Floriculture and Dendrology, Faculty of Horticultural Science, Szent István University, Villányi út 29-43, 1119 Budapest, Hungary; dioszegi.magdolna@kertk.szie.hu; 7 National Biosafety Laboratory, National Public Health Center, Albert Flórián út 2-6, 1097 Budapest, Hungary; palyi.bernadett@nnk.gov.hu (B.P.); kis.zoltan@oki.antsz.hu (Z.K.)

**Keywords:** *Forsythia*, *Plantago*, forsythoside, plantamajoside, acteoside, isomerization, Vero cells

## Abstract

A comparative phytochemical study on the phenylethanoid glycoside (PhEG) composition of the underground organs of three *Plantago* species (*P. lanceolata*, *P. major,* and *P. media*) and that of the fruit wall and seed parts of *Forsythia suspensa* and *F. europaea* fruits was performed. The leaves of these *Forsythia* species and six cultivars of the hybrid *F. × intermedia* were also analyzed, demonstrating the tissue-specific accumulation and decomposition of PhEGs. Our analyses confirmed the significance of selected tissues as new and abundant sources of these valuable natural compounds. The optimized heat treatment of tissues containing high amounts of the PhEG plantamajoside (PM) or forsythoside A (FA), which was performed in distilled water, resulted in their characteristic isomerizations. In addition to PM and FA, high amounts of the isomerization products could also be isolated after heat treatment. The isomerization mechanisms were elucidated by molecular modeling, and the structures of PhEGs were identified by nuclear magnetic resonance spectroscopy (NMR) and high-resolution mass spectrometry (HR-MS) techniques, also confirming the possibility of discriminating regioisomeric PhEGs by tandem MS. The PhEGs showed no cytostatic activity in non-human primate Vero E6 cells, supporting their safe use as natural medicines and allowing their antiviral potency to be tested.

## 1. Introduction

To determine the novel plant sources of valuable phenylethanoid glycosides (PhEGs) showing no cytotoxicity against normal cells [1], we investigated various tissues of *Plantago* and *Forsythia* species.

From the large genus of *Plantago* (order Lamiales, family Plantaginaceae), comprising about 200 species, *Plantago lanceolata* L. (ribwort plantain), *P. major* L. (broadleaf plantain), and *P. media* L. (hoary plantain) were involved in this study. Among them, *P. lanceolata* and *P. major* are widely distributed around the world, while *P. media* is common in Europe and Asia but also locally in the USA [2]. These herbaceous perennial plants develop an underground rhizome that bears roots, a solitary, erect flower stem, and leaves forming a rosette [3]. Among these three plant species, *P. lanceolata* and *P. major* are amongst the most commonly used medicinal herbs in the world. Various extracts of their aerial parts exhibit many beneficial effects on human health, including wound-healing, anti-inflammatory, antioxidant, and chemopreventive effects [2]. Based on the therapeutic significance of the *P. lanceolata* leaf, it is listed as an official drug in the European Pharmacopoeia (Ph. Eur., 10th edition). Furthermore, the leaves of *P. lanceolata* [4] and *P. major* [5] are edible and can be used as vegetables. Despite being readily available in the nature, these two plantain species are also crops cultivated in many countries [4,6].

The genus *Forsythia* (order Lamiales, family Oleaceae) consists of about 10 species native to eastern Asia, with the single exception of *F. europaea* Degen & Bald, which is native to southeastern Europe. *Forsythia* plants are deciduous shrubs with opposite leaves and yellow flowers blooming in early spring before the appearance of leaves. The fruit, if formed, is a capsule that, at maturity, splits open to release its numerous seeds [7]. Two *Forsythia* species, *F. suspensa* Vahl and *F. viridissima* Lindl., and particularly their hybrid *F. × intermedia*, are widely cultivated as ornamentals for their flowers in temperate zones worldwide. On the contrary, *F. europaea* is not commonly cultivated and can only be found in special plant collections outside its habitat [7]. In our study, among the *Forsythia* species, *F. europaea*, *F. suspensa*, and *F. × intermedia*, which are also available in botanical gardens in Europe, were studied. The fruits and leaves of *F. suspensa* have been used for centuries in eastern medicine: the fruits as anti-inflammatory and diuretic agents and the leaves as health tea for colds [8]. The fruits are also listed as an official drug in the Chinese, Japanese, and Korean Pharmacopoeias [9].

**Figure 1:**
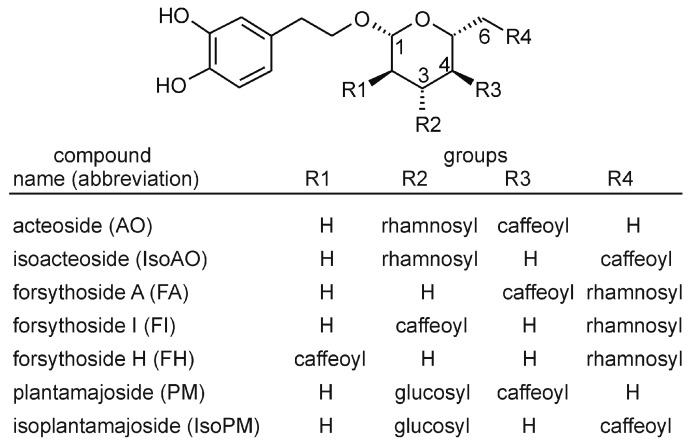
Chemical structures of phenylethanoid glycosides analyzed in *Plantago* and *Forsythia* plants.

The PhEG acteoside (AO) is a chemotaxonomic marker compound of the order Lamiales [10]. Thus, this compound is also present in many species of the genera *Plantago* and *Forsythia*. Structurally, AO consists of a centrally positioned β-D-glucose that is linked at its C1, C3, and C4 hydroxyl groups to hydroxytyrosol, α-L-rhamnose, and caffeic acid units, respectively. Some Lamiales plants also contain (i) isomers of AO, such as isoacteoside (IsoAO), forsythoside A (FA), forsythoside H (FH), and forsythoside I (FI), whose rhamnosyl and caffeoyl units are linked at C3 and C6 (IsoAO), C6 and C4 (FA), C6 and C2 (FH), or C6 and C3 (FI) positions, and (ii) closely related compounds of AO, such as plantamajoside (PM) and isoplantamajoside (IsoPM), which contain a glucosyl unit instead of the rhamnosyl units of AO and IsoAO, respectively (Figure 1).

Representatives of these PhEGs were confirmed to be characteristic and active compounds for our selected plants, i.e., PM, AO, and IsoAO in *Plantago* species [2] and FA, FH, FI, AO, and IsoAO in *Forsythia* species [11,12,13]. 

As to the accumulation of AO in Lamiales plants, our research group determined its high levels in different tissues of *Fraxinus* [1], *Euphrasia* [14], *Syringa* [15], and *Olea* [16] plants. The isolation of a significant amount of pure AO can be performed easily from these plant tissues through a one-step preparative high-performance liquid chromatography (HPLC) separation. Optimized heat treatment of AO, performed in distilled water, resulted in its isomerization into IsoAO, forming an equilibrium mixture of AO and IsoAO. The high-yield isolation of IsoAO can also be performed from this mixture through preparative HPLC [1]. 

Since AO and IsoAO are already readily available, we focused on the analysis and isolation of PM, IsoPM, FA, FH, and FI being present in *Plantago* and *Forythia* plants. Plantamajoside has been identified in 34 plant species belonging to the order Lamiales [17]. The highest amounts of PM were detected in the root cultures of *P. lanceolata* [18] and in the roots of *P. lanceolata* [19] and *P. media*, which were grown in vitro [20]. However, no data are available regarding the PhEGs of underground organs of wild-grown *Plantago* species. Isoplantamajoside is a rarely occurring PhEG, which is determined as a minor compound only in five Lamiales species, including one representative of the genus *Plantago* (*P. asiatica*) [21].

When analyzing the PhEG composition of *Forsythia* plants, FA was determined to be the main compound in the fruits of *F. suspensa* and *F. europaea* as well as in the leaves *of F. suspensa* and *F. × intermedia* [11,22,23,24]. However, in the leaves of *F. europaea*, FA [11,25] and AO [26] were also confirmed as the main PhEGs. Two isomers, FH and FI, were determined as minor metabolites being present exclusively in the fruit of *F. suspensa* [12,27]. In addition to PhEGs, *Forsythia* species also accumulate the flavonoid glycoside rutin [22,23,24,28]. 

The identification of PhEGs was mainly based on their multistep isolation followed by spectroscopic analyses. However, the isomers, which can be identified using identical molecular formulas C_29_H_36_O_16_ (PM and IsoPM) or C_29_H_36_O_15_ (AO, IsoAO, FA, FH, and FI), were not distinguished from each other by their mass fragmentation properties. Furthermore, there is no HPLC method for the complete separation of all isomers AO, IsoAO, FA, FH, and FI, being present side by side. Consequently, the on-line HPLC-MS identification of PhEG isomers remains incomplete. 

The effect of heat treatment on isolated FA in a water medium has been analyzed by HPLC-MS, showing the characteristic conversion of FA into FH and FI [29]. Considering this result, a heat treatment of selected tissues containing FA may allow the high-yield isolation of FH and FI; however, the identity of the conversion products FH and FI needs to be confirmed.

The main PhEG compounds of *Plantago* and *Forsythia* plants might be responsible, at least partly, for their medicinal activity detailed above. Accordingly, the wound healing activity of PM and anti-inflammatory activity of FA were confirmed. Furthermore, their antiproliferative, antibacterial, and antioxidant properties have also been described [17,30]. The bioactivity of the minor PhEGs IsoPM, FH, and FI has been less frequently studied due to their limited availability. The antioxidant properties of these metabolites [31,32,33] as well as the antibacterial effects of FH and FI [31,32], and the antihypertensive [34] and cardioprotective [35] effects of IsoPM, have already been confirmed. The remarkable antiviral activity of FA against influenza [36] and avian bronchitis viruses [37], and that of AO against the respiratory syncytial virus [38] and Dengue virus 2 [39] has also been confirmed, highlighting the relevance of testing all PhEGs for their antiviral potency against other viruses such as SARS-CoV-2. However, prior to performing further efficacy studies with these PhEGs, their non-toxicity against normal cell lines should also be investigated. 

The aims of this research are as follows:

(1) Determine the PhEG composition in the underground organs (i.e., in roots and rhizomes) of wild-grown *P. lanceolata*, *P. major*, and *P. media*,
(2) Compare the PhEG profiles in the leaves of *F. × intermedia* (represented by six cultivars), *F. suspensa*, and *F. europaea* and follow the accumulation of PhEGs in the separated fruit parts (i.e., fruit wall and seed) of *F. suspensa* and *F. europaea* during fruit ripening with an ultra high-performance liquid chromatography (UHPLC)-MS method allowing the complete, baseline separation of AO, IsoAO, FA, FH, and FI,
(3) Conduct a mass fragmentation study of PhEGs to distinguish the isomers by their MS/MS profiles,
(4) Develop a heat treatment procedure for PM and FA conversion to obtain their isomers at the highest yield possible, allowing the isolation of IsoPM (from selected *Plantago* tissues) as well as FH and FI (from selected *Forsythia* tissues) through one-step preparative HPLC,
(5) Confirm the structures of the conversion products by NMR and computational calculations interpreting the isomerization processes,
(6) Confirm the practical utility of the selected *Plantago* and *Forsythia* tissues in the high-yield isolation of PhEGs, and
(7) Determine the in vitro toxicity of all isolated PhEGs against normal cells (Vero E6).

## 2. Results and Discussion

### 2.1. Identification of Compounds

**Figure 2:**
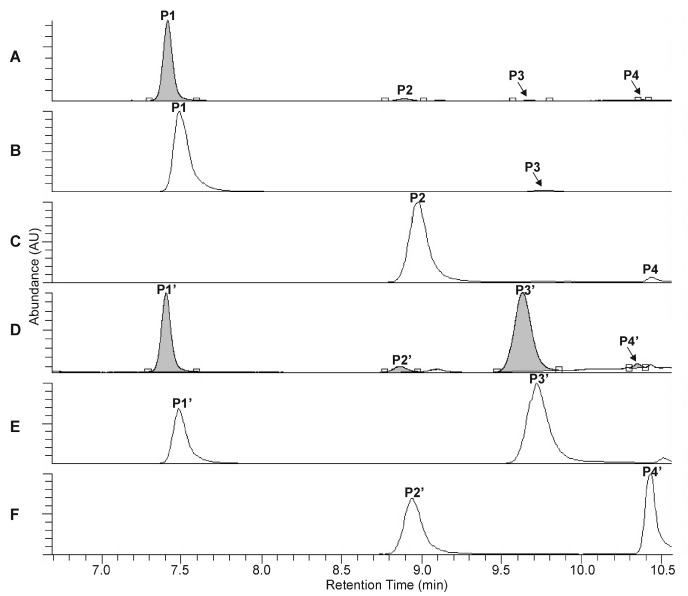
UHPLC-UV (λ = 330 nm) chromatograms (**A**,**D**) and extracted ion chromatograms (**B**,**C**,**E**,**F**) for *m*/*z* 639 (**B**,**E**) and *m*/*z* 623 (**C**,**F**) corresponding to the phenylethanoid glycosides plantamajoside (**P1**, **P1′**), isoplantamajoside (**P3**, **P3′**), acteoside (**P2**, **P2′**), and isoacteoside (**P4**, **P4′**), which were obtained from the intact *P. major* rhizome sample (**A**–**C**) and from the *P. major* rhizome sample after 120 min of heat treatment (**D**–**F**).

**Figure 3:**
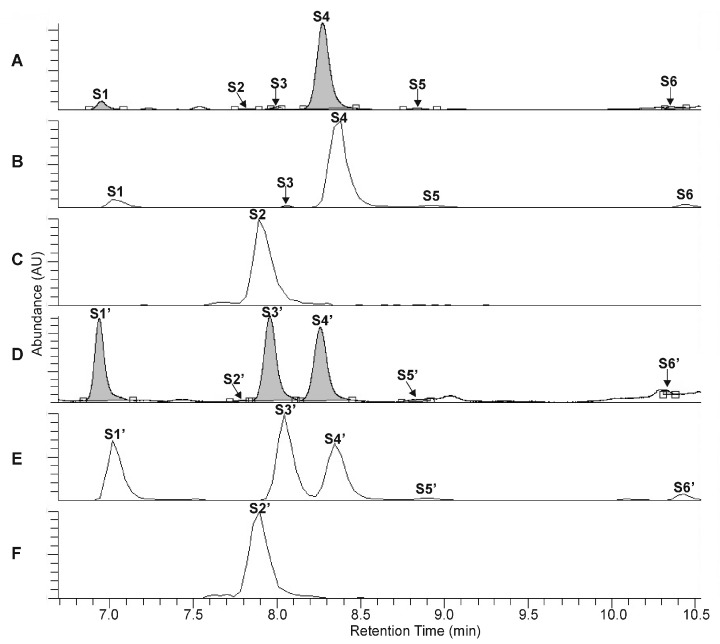
UHPLC-UV (λ = 330 nm) chromatograms (**A**,**D**) and extracted ion chromatograms (**B**,**C**,**E**,**F**) for *m*/*z* 623 (**B**,**E**) and *m*/*z* 609 (**C**,**F**) corresponding to the phenylethanoid glycosides forsythoside I (**S1**, **S1′**), forsythoside H (**S3**, **S3′**), forsythoside A (**S4**, **S4′**), acteoside (**S5**, **S5′**) isoacteoside (**S6**, **S6′**), and the flavonoid glycoside rutin (**S2**, **S2′**), obtained from the intact unripe fruit wall sample of *F. suspensa* (**A**–**C**) and from the unripe fruit wall sample of *F. suspensa* after 300 min of heat treatment (**D**–**F**).

The UHPLC-UV-HR-MS separation of the extracts, prepared from the rootstock of *P. major* and from the unripe fruit wall of *F. suspensa*, confirmed the presence of four compounds in *Plantago* (**P1**–**P4**, Figure 2A–C) and six compounds in *Forsythia* (**S1**–**S6**, Figure 3A–C) samples.

Based on their HR-MS data, identical molecular formulas were determined for **P1** and **P3** (C_29_H_36_O_16_) as well as for **P2**, **P4**, **S1**, and **S3**–**S6** (C_29_H_36_O_15_) (Table 1).

Compound **S2** can be identified using the molecular formula C_27_H_30_O_16_. The UHPLC chromatograms show **P1** and **S4** to be most abundant compounds in the *Plantago* and *Forsythia* extracts, respectively (Figure 2A–C and Figure 3A–C). Comparing molecular formulas, elution properties, and quantitative relationship data with those obtained for already identified compounds of *Plantago* and *Forsythia* species, four PhEGs [PM (**P1**) IsoPM (**P3**), AO (**P2**), and IsoAO (**P4**)] in *Plantago* extract and five PhEGs [FI (**S1**), FH (**S3**), FA (**S4**), AO (**S5**), and IsoAO (**S6**)] and a flavonoid glycoside [rutin (**S2**)] in the *Forsythia* extract, were presumed to be present. Standard AO, IsoAO (isolated recently in our laboratory, [1]), and rutin (purchased from Sigma) were also analyzed by UHPLC-HR-MS/MS, resulting in comparable mass fragmentation and retention properties to those for compounds **P2**/**S5**, **P4**/**S6**, and **S2**, respectively. This result confirms the identity of **P2** and **S5** as AO, **P4** and **S6** as IsoAO, and **S2** as rutin.

Conversion studies were conducted to confirm the structures of other PhEGs. Tissue suspensions, prepared from powdered plant tissues (i.e., rootstock of *P. major* and unripe fruit walls of *F. suspensa*) by adding water, heated at 100 °C for 300 min, and analyzed by UHPLC-UV-HR-MS. The main compounds in the intact tissues of *P. major* (PM, Figure 2A, peak **P1**) and *F. suspensa* (FA, Figure 3A, peak **S4**) showed characteristic conversions during the heating procedure. Heat treatment resulted in the formation of one compound from PM (Figure 2D, peak **P3′**) and two compounds from FA (Figure 3D, peaks **S1′** and **S3′**). The conversion products and their corresponding precursors could be characterized by identical molecular formulas, which were determined as C_29_H_36_O_16_ for **P3**′ and PM and C_29_H_36_O_15_ for **S1′**, **S3′**, and FA. These data confirm an isomeric relationship between PM and its conversion product **P3′**, as well as between FA and its conversion products **S1′** and **S3′**. In addition to computational calculations interpreting the isomerization of PM into **P3′** and FA into **S1**′ and S3′ (Section 2.5), NMR analyses and mass fragmentation studies of these isolated PhEGs (as detailed below) confirmed the structures of the conversion products **P3′**, **S1′**, and **S3′** as IsoPM, FI, and FH, respectively, and their corresponding precursors as PM and FA. Based on these results, compounds appearing with comparable retentions in the chromatograms of intact extract and heat-treated extracts were confirmed to be identical. 

Our results confirm for the first time (1) the convertibility of PM into IsoPM and (2) the isomerization of FA into FH and FI that takes place in plant tissue containing FA suspended in a hot water medium. Additional NMR spectral data for PM, IsoPM, FA, FI, and FH, isolated from their optimum sources, were comparable to that reported in the literature for these PhEGs, thereby unambiguously confirming their identity (Supplementary material, NMR data) [12,21,40]. 

Fragment ion spectra of PM, IsoPM, FA, FI, FH, AO, and IsoAO, generated from the deprotonated molecular ions of these compounds (i.e., from ion *m/z* 623 of FA, FI, FH, AO, and IsoAO and from ion *m/z* 639 of PM and IsoPM) using various collision-induced dissociation (CID) energies (ranging from 20 to 35 eV) were also compared. In accordance with the literature [1,22,23,41], identical fragment ions were confirmed for isomers FA, FI, FH, AO, and IsoAO as well as for isomers PM and IsoPM (Table 2). However, the difference in the relative ion intensities of two key fragment ions *m/z* 461 and *m/z* 179, corresponding to the deprotonated hydroxytyrosol–rhamnosylglucoside and caffeic acid moieties, allowed the discrimination between forsythoside isomers (FA, FI, FH) and acteoside isomers (AO, IsoAO). The abundance ratios between ions *m/z* 461 and *m/z* 179 were calculated to be less than 1 for forsythoside isomers (0.80, 0.54, and 0.97 for FA, FI, and FH, respectively) whilst exceeding 5 for acteoside isomers (5.42 for AO and 5.97 for IsoAO) (Table 2).

Furthermore, the members of the isomer pairs AO–IsoAO and PM–IsoPM can also be discriminated, as the relative intensities of the deprotonated molecular ions in the spectra of IsoAO and IsoPM were significantly higher than those of the spectra of AO and PM. Namely, the intensity ratios of the ion *m/z* 623 between IsoAO and AO and that of the ion *m/z* 639 between IsoPM and PM, were calculated as being 2.34 (Table 2, 13.9 divided by 5.94) and 2.63 (Table 2, 33.7 divided by 12.8), respectively. These data confirm that IsoPM and IsoAO are more stable against fragmentation processes generated by CID than PM and AO.

By analyzing the elution profiles of PhEG isomers FA, FH, FI, AO, and IsoAO characterized by identical *m/z* values, we have confirmed for the first time that these PhEGs can be separated from each other using our RP-HPLC method (Supplementary Material Figure S1).

### 2.2. PhEG Composition in the Underground Organs of Plantago Plants

The underground organs of several year-old *Plantago* plants can be separated into rhizome and root parts by manual dissection, allowing the comparison of their PhEG compositions. Since the rhizomes of young plants are undeveloped, only their root systems were analyzed. The PhEG PM was determined as the main compound in the underground organs of *P. major*, reaching its highest level in the rhizomes (Table 3). 

The average PM content of these rhizomes (76.6 mg/g) corresponds to that reported as being the highest in the plant kingdom (30–80 mg/g, determined in the in vitro root culture of *P. lanceolata*) [18], thus highlighting the significance of *P. major* rhizomes in the production of this valuable natural PhEG. The amounts of AO were significantly higher than those of PM in all analyzed underground organ samples of *P. lanceolata* and *P. media*. However, previous studies have confirmed the dominance of PM in the roots of these plants when grown in vitro [19,20]. Since the PhEG biosynthesis could be influenced by various stress stimuli (e.g., microbial attack) [18], we assume that interactions between wild-grown *Plantago* roots and microorganisms result in the change in the quantitative ratio of AO and PM, which are characteristic for roots grown in vitro under sterile conditions.

### 2.3. PhEG Composition of Forsythia Plants

The hybrid *F. × intermedia* is known to have many precisely defined cultivars (e.g., ‘Lynwood’, ‘Melisa’, ‘Minigold’, ‘Primulina’, ‘Spectabilis’, and ‘Week End’). As *F. × intermedia* cultivars generally do not form fruits in temperate climates, more attention has been paid to the analysis of their leaf compounds [42]. However, only unspecified cultivars have been previously analyzed, confirming the co-occurrence of the PhEG compounds FA and AO in their leaves [11,42]. The ratios of the amounts FA and AO, calculated from HPLC quantitation data (49 mg/g FA and 15 mg/g AO, dry weight) [11] and isolated yields (0.16 mg/g FA and 0.11 mg/g AO, dried leaf) [42], were 3.3 (49 mg/g FA divided by 15 mg/g AO = 3.3) and 1.5 (0.16 mg/g FA divided by 0.11 mg/g AO = 1.5), respectively. We compared the metabolite composition in the leaves of six specified *F. × intermedia* cultivars, confirming for the first time that the PhEGs FA and AO were the main compounds in the leaves of each cultivar (Supplementary Material, Table S1). The amounts of the main PhEGs FA and AO were found to be highly variable between the cultivars as well as between individuals of the same cultivars. However, the ratios of the amounts FA and AO in the leaf samples proved to be nearly constant, varying between 1.5 (cultivar ‘Melisa’, individual 1, 43.7 mg/g FA divided by 28.4 mg/g AO = 1.5) and 2.7 (cultivar ‘Primulina’, individual 2, 66.4 mg/g FA divided by 24.6 mg/g AO = 2.7). These ratios of FA and AO are in good accordance with those obtained from the literature (3.3 and 1.5, as detailed above), indicating that the dominance of FA over AO is characteristic of the leaves of *F. × intermedia* cultivars (Supplementary material, Table S1). 

The literature data show that the main PhEG compound in the leaf and fruit samples of *F. europaea* collected from two Far Eastern and one European botanical garden is FA (in the Far Eastern leaf and fruit samples) [11,43] or AO (in the European leaf sample) [26]. The results of our study performed on Hungarian *F. europaea* leaves (Supplementary material, Table S1) and fruits (as detailed below) confirmed AO as the main compound in these organs. These data suggest a chemical diversity between *F. europaea* plants, living in different habitats, as FA and AO were found to be the main compounds in the Far Eastern and European *F. europaea* plant tissues, respectively. 

Based on the literature results, FA was determined as being the main PhEG compound in the fruit and leaf samples of *F. suspensa*. Great attention was paid to the determination of PhEGs accumulated by the unripe and ripe fruits of *F. suspensa*. The FA contents of the unripe fruits were 5 or 15 times higher than those of the ripe fruits [23,24]. Furthermore, high levels of FA were determined in the separated seeds obtained from the unripe fruits of *F. suspensa* (47 mg/g, dry weight) [23], highlighting the need to follow PhEG accumulation in both separated fruit parts (i.e., fruit wall and seed) of *F. suspensa* and *F. europaea* during fruit ripening. In accordance with previous results [11,22], our *F. suspensa* leaf samples contained high levels of FA (88.3 mg/g, dry weight, average of leaf samples collected from two individuals) (Supplementary material, Table S1). While analyzing the metabolite composition of both fruit parts (i.e., seed and fruit wall) during the fruit ripening of *F. europaea* and *F. suspensa*, a fruit part specific accumulation and decomposition of PhEGs was confirmed for the first time. In fact, the unripe fruit walls of *F. europaea* and *F. suspensa* accumulated high amounts of AO (71.4 mg/g, dry weight) and FA (80.4 mg/g, dry weight), respectively (data are the average of unripe fruit wall samples (StA) collected from two individuals) (Table 4).

However, the amounts of these PhEGs were negligible in the corresponding fruit walls obtained from the fully ripe fruits (marked with StC in Table 4), confirming that the decomposition of AO and FA takes place in the fruit walls during their ripening processes. In addition to the unripe fruit wall of *F. suspensa*, the seeds of this plant, isolated from the unripe and ripe fruits, were also rich in FA (ripe seed: 61.7 mg/g, marked with StC in Table 4; unripe seed: 58.7 mg/g, marked with StA in Table 4, average data of seed samples collected from two individuals). Thus, FA was found to be stable in the seeds of *F. suspensa* during fruit ripening. Based on these results and taking into account that (1) *Forsythia* fruits consist of nearly equal amounts of fruit wall and seed parts, and (2) the ripe fruits split open to release their seeds, we can explain the differences between the ratios of the FA amounts determined in the unripe and ripe fruits of *F. suspensa* (literary results, as detailed above: 5 vs. 15 times higher FA contents in the unripe fruit than in the ripe fruits) [23,24]. 

Based on our results, the FA content ratios between the unripe and ripe fruits should be a relatively small value if all the FA-bearing seeds remained in the ripe fruit. We calculated this ratio to be 2.2. However, the value of this ratio might also be extremely high, reaching a value of around 40, as the result of a complete seed loss.

### 2.4. Practical Utility of Plantago and Forsythia Tissues in the Isolation of PhEGs

The PhEGs IsoPM, FH, and FI were minor metabolites in the intact tissues investigated (Table 3 and Table 4 and Supplementary Material Table S1), and thus, their isolation from these tissues is a challenging task, requiring multistep purification procedures and the extraction of high amounts of plant tissues. However, from selected intact *Plantago* and *Forsythia* tissues containing high amounts of PM and FA, the other related minor PhEGs, IsoPM, FH, and FI, can also be prepared in high yield, using a heat treatment procedure followed by one-step preparative HPLC isolation. However, optimum starting tissues and treatment periods need to be defined to avoid the presence of contaminants that are not separable from PhEGs, prepare conversion products at the highest yield possible, and minimize the unwanted decomposition of PhEGs. Based on our comparative analysis of *Plantago* and *Forsythia* tissues, *P. major* rhizomes (marked with collection number 2 in Table 3) and *F. suspensa* unripe fruit walls (marked with collection number 1 of sample StA in Table 4) were selected due to their extremely high PM and FA contents, respectively, to isolate PM, FA, and their corresponding isomers after an optimized heat treatment procedure. The leaves of *F. suspensa* also contained extremely high amounts of FA (88.3 mg/g, as detailed above) along with remarkable levels of rutin (5.22 mg/g, dry weight, average of leaf samples collected from two individuals) (Supplementary Material, Table S1). The flavonoid glycoside rutin and the PhEG FH were identified by UHPLC as being close-eluting compounds and thus, FH isolated by one-step preparative HPLC may contain rutin as an impurity. An additional reason for selecting *F. suspensa* unripe fruit walls was their low rutin content measured among *Forsythia* samples (0.67 mg/g, dry weight, average of fruit wall samples collected from two individuals, Table 4), allowing the isolation of FH with the lowest possible amount of rutin impurities.

**Figure 4:**
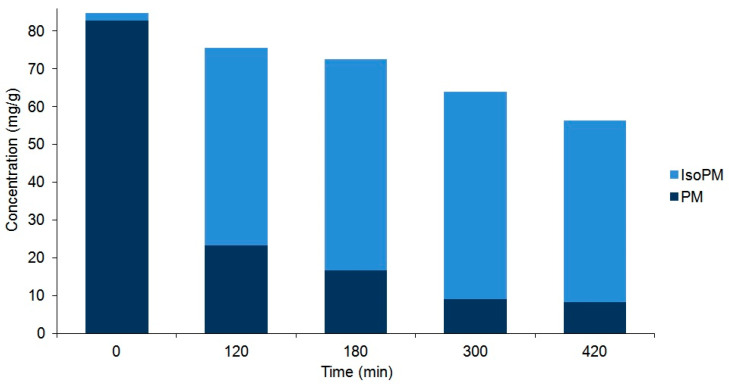
Comparison of the compositions of plantamajoside (PM) and isoplantamajoside (IsoPM) in the rhizome samples of *P. major* that were heated for different periods of time (from 0 to 420 min). Values are the averages of three parallel heat treatments. Differences could be characterized by the relative standard deviation (RSD) values, ranging from 3.7% (PM in unheated intact 0 min sample) to 10.1% (PM in sample heated for 420 min). Corresponding chromatograms of unheated sample and those of samples heated for 120 min are shown in Figure 2.

**Figure 5:**
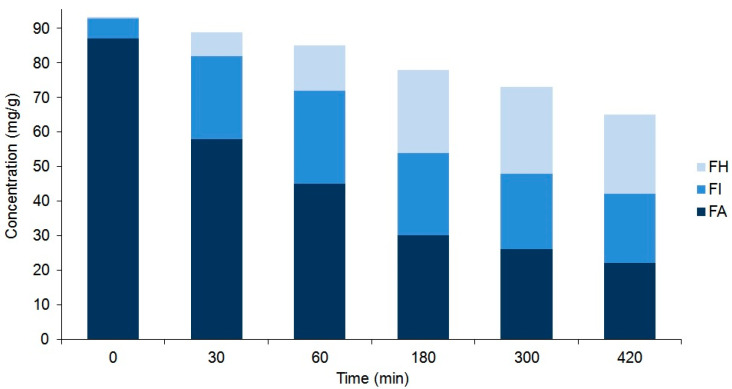
Comparison of the composition of forsythoside A (FA), forsythoside H (FH), and forsythoside I (FI) in the unripe fruit wall samples of *F. suspensa* that were heated for different periods of time (from 0 to 420 min). Values are the averages of three parallel heat treatments. Differences could be characterized by the relative standard deviation (RSD) values, ranging from 3.4% (FA in unheated intact 0 min sample) to 11.0% (FH in sample heated for 420 min). Corresponding chromatograms of unheated samples and those of samples heated for 300 min are shown in Figure 3.

Accordingly, tissue suspensions, prepared from these selected powdered plant tissues (i.e., the rootstock of *P. major* and unripe fruit wall of *F. suspensa*) by adding water, were heated at 100 °C for different periods of time and analyzed by UHPLC-UV-HR-MS. As detailed in the previous Section 2.1, the heating of these suspensions resulted in the conversion of PM and FA into their corresponding isomers IsoPM, FI, and FH. Heat treatments performed for different periods of time confirmed a simultaneous isomerization and undefined degradation processes of PhEGs (Figure 4 and Figure 5).

As a result of these processes, an equilibrium mixture of IsoPM and PM was formed (with 85% IsoPM and 15% PM contents) as well as that of FI, FH, and FA (with 31% FI, 35% FH, and 34% FA contents) (Figure 4 and Figure 5). These results are in agreement with data obtained using the mass fragmentation analysis and computational methods (as detailed in Section 2.1 and Section 2.5), indicating the greater stability of IsoPM than PM and a comparable stability of forsythoside isomers (FA, FH, and FI). These equilibria were formed after 300 min of heat treatments of the *Plantago* and *Forsythia* samples. Longer heat treatments resulted in undefined degradations of all PhEGs without any effects on equilibrium compositions. 

Consequently, to isolate the minor PhEGs IsoPM, FI, and FH at the highest yield possible by preparative HPLC, the suspensions of *P. major* rhizomes and *F. suspensa* unripe fruit walls were heated for 300 min. Thus, starting from 1.0 g plant tissues (i) 24.0 mg IsoPM of 86% purity from *P. major* rhizome and (ii) 4.6 mg FI and 5.6 mg FH of 96% and 85% purity, respectively (and 3.6 mg FA of 92% purity) from the *F. suspensa* unripe fruit wall, were isolated (Supplementary Material, Figure S1). A review of the contents of these PhEGs among plants showed that 0.67 mg/g of IsoPM (in *P. asiatica*, whole plant [21]), 0.22 mg/g of FI, and 0.012 mg/g of FH (in *F. suspensa*, ripe fruit [27]) were the highest amounts recorded. The amounts of IsoPM, FI, and FH isolated after optimized heat treatments were 36, 21, and 470 (!) times higher than those reported to date as being the highest for these compounds in the plant kingdom. Since the equilibrium mixture, prepared through 300 min heating of the *P. major* rhizome suspension, contained a relatively small amount of PM (15% PM vs. 85% IsoPM), the untreated intact *P. major* rhizome was used to isolate PM. Thus, 56.1 mg PM of 96% purity could be isolated from 100.0 mg of the *P. major* rhizome. 

These results highlight the significance of selected plant tissues, i.e., *F. suspensa* unripe fruit walls and *P. major* rhizomes, in the isolation of PhEGs (FA, FH, FI, PM, and IsoPM) after optimized heat treatments, as easily available, new raw materials for these pharmacologically important metabolites.

As a further result of our comparative analysis of the *Plantago* and *Forsythia* tissues, a high-level accumulation of AO was also detected. The average AO contents in the underground organs of *P. media* (78.1 mg/g, Table 3) and in the leaves (73.6 mg/g, Supplementary material, Table S1) and unripe fruit walls (sample StA: 71.4 mg/g, Table 4) of *F. europaea* are the highest reported in the plant kingdom [1], thus highlighting the significance of these plant tissues in the production of the valuable PhEG-type metabolite AO.

### 2.5. Computational Modeling of the Isomerization of PM into IsoPM and FA into FI and FH

In our previous study, the isomerization of AO into IsAO in a hot water medium was confirmed as an intramolecular acyl transfer reaction [1]. The in silico study of the thermodynamic (driving force) and kinetic (reaction rate) aspects of this transformation confirmed a slightly greater stability of IsoAO relative to AO. This computational result explained the formation of the equilibrium mixture consisting of 54% IsoAO and 46% AO, which was determined after heating of AO in a water medium [1]. 

The isomerization of PM into IsoPM and that of FA into FI and FH is also an intramolecular acyl transfer reaction, involving the migration of the caffeoyl ester moiety from the C4 position to the close hydroxyl groups (Supplementary Material, Figures S2 and S3) [44,45,46]. Both the thermodynamic (driving force) and kinetic (reaction rate) aspects of these transformations were studied by theoretical methods at the B3LYP/6-31G(d,p) level of theory [47] with an IEFPCM solvent model [48] by Gaussian 16 [49]. From a thermodynamic point of view, the transformation of PM into IsoPM is a significantly exothermic process, as the enthalpy (Δ*H*) and Gibbs free energy (Δ*G*) decrease by –24.2 kJ mol^–1^ and –26.2 kJ mol^–1^, respectively. These values indicate a definite conversion process toward IsoPM, which is also supported by the beneficial entropy change (Δ*S*, +6.7 J mol^–1^ K^–1^) (Supplementary Material, Figure S2). This entropy factor can originate from the formation of a sterically less hindered, more freely rotating side chain. Another isomer can also be considered as a possible product of PM isomerization (Supplementary Material, Figure S2, IsoPM-2). However, in this case the computed enthalpy value is significantly endothermic, thus excluding the existence of this product. From a thermodynamic point of view, the transformations of FA into FI and FI into FH are almost thermoneutral, as the enthalpy and Gibbs free energy changes are near zero (Supplementary Material, Table S2). These values refer to a chemical equilibrium between the three isomers. 

Acyl transfer reactions can be characterized by the change of the carbonylicity percentage (CA%), where the increase of this value (ΔCA% > 0) during the reaction implies a beneficial exothermic process [44,45,46]. The comparable CA% values of PM (52.2%) and IsoPM (52.9%) as well as those of FA (52.9%), FI (53.0%), and FH (52.5%) confirm the absence of an internal driving force for the transformations (Supplementary Material, Figure S2, Table S2). 

We also studied the reaction mechanisms of PM and FA isomerizations by an explicit–implicit solvent model (Supplementary Material, Figure S4, Table S3) [50]. This model confirmed that these isomerization processes consist of two elementary steps, allowing a relatively fast reaction rate at elevated temperature (Supplementary Material, Table S3).

These computed data support the isomer proportions of 85:15 for IsoPM and PM, and that of 31:35:34 for FI, FH, and FA, determined experimentally in a water medium after 300 min of heating.

### 2.6. In Vitro Activity of Isolated PhEGs on Non-Human Primate Vero E6 cells

**Figure 6:**
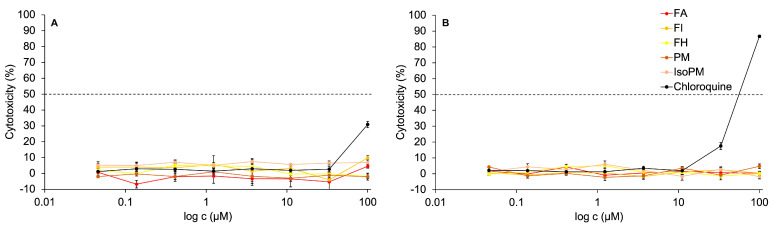
In vitro activity of the isolated phenylethanoid glycosides forsythoside A (FA), forsythoside H (FH), forsythoside I (FI), plantamajoside (PM), and isoplantamajoside (IsoPM) and that of the positive control chloroquine on Vero E6 cells after treatment periods of 24 h panel (**A**) and 48 h panel (**B**).

Recently, the non-toxicity of PM on hamster ovary cells [51] and FA on canine kidney [36] and chicken embryo kidney cells [37] has been confirmed. However, IsoPM, FH, and FI had not yet been tested against normal healthy cells such as kidney epithelial cells isolated from *Cercopithecus aethiops* (Vero E6). Thus, the in vitro activity of PM, IsoPM, FA, FH, and FI, isolated from their optimum sources, was analyzed on Vero E6 cells for the first time. In the concentration range tested (0.04–100 μM), none of these PhEGs expressed cytotoxicity on Vero E6 cell cultures. In contrast, treating Vero E6 cells with 100 μM chloroquine for 24 h resulted in 30% cytotoxicity, while after a 48 h treatment, most of the cells died (cytotoxicity = 86%) at this concentration (Figure 6).

To perform in vitro antiviral tests, Vero E6 cells are often used as a host cell model for growing viruses. In this model, promising antiviral drug candidates should not exhibit cytotoxicity on Vero E6 cells. Consequently, based on our results confirming the absence of cytotoxicity of the PhEGs on Vero E6 cells, the antiviral tests of PhEGs against the SARS-CoV-2 virus can be performed using Vero E6 cells.

## 3. Materials and Methods

### 3.1. Plant Material and Reagents

The underground organs of three *Plantago* species (*P. lanceolata* L., *P. major* L., and *P. media* L.) were collected from different Hungarian locations in June of 2018. Leaf and fruit samples of the *Forsythia* species (*F. europaea* Degen & Bald, *F. suspensa* Vahl, and *F. × intermedia* cultivars ‘Lynwood’, ‘Melisa’, ‘Minigold’, ‘Primulina’, ‘Spectabilis’, and ‘Week End’) were collected from the Botanical Garden of Szent István University (Budapest, Hungary, Villányi street 29–43): the leaves and unripe, green fruits in July of 2018 and the ripe, yellow-brown, closed fruits as well as the ripe, yellow-brown, opened fruits in October of 2018. The collected samples were immediately lyophilized on the day of collection. The voucher specimens of all dried samples are deposited in the Department of Plant Anatomy, Eötvös Loránd University, Budapest, Hungary. The materials and reagents applied in the analysis and isolation of plant metabolites, such as acetonitrile (ACN), distilled water (DW), formic acid, dimethyl sulfoxide (DMSO), methanol (Reanal, Budapest, Hungary), DMSO-*d*_6_, and methanol-*d*_4_ (VWR chemicals, Leuven, Belgium) were all of analytical reagent grade of the highest purity available. 

### 3.2. Performing Heat Treatments

Lyophilized and pulverized plant tissues (100.0 mg) were suspended in 2.0 mL of DW in 5 mL screw-capped vials. These suspensions were heated at 100 °C for 30, 60, 120, 180, 300, and 420 min. After heating, the samples were lyophilized.

### 3.3. Preparation of Plant Extracts

Lyophilized and pulverized intact plant tissues (100.0 mg), as well as heat-treated and lyophilized tissues, were extracted three times consecutively with 5 mL of methanol at 60 °C in 25 mL screw-capped vials for 30 min to prepare 15.0 mL stock solutions. These stock solutions were used after their dilution with methanol for UHPLC-UV-HR-MS analyses (Section 3.4.1). Dried stock solutions, prepared from the intact and from the 300 min heat-treated *P. major* rhizome samples (marked with collection number 2) and those from the 300 min heat-treated *F. suspensa* unripe fruit wall sample (marked with collection number 1), were dissolved in 2.0 mL of methanol for the isolation of PM, IsoPM, and forsythoside isomers (FA, FI, FH) by preparative HPLC, respectively (Section 3.4.2).

### 3.4. Instruments 

#### 3.4.1. Analytical UHPLC Hyphenated with UV and High-Resolution Orbitrap Mass Spectrometric Detections 

A Dionex Ultimate 3000 UHPLC system (3000RS diode array detector (DAD), TCC-3000RS column thermostat, HPG-3400RS pump, SRD-3400 solvent rack degasser, WPS-3000TRS autosampler), hyphenated with an Orbitrap Q Exactive Focus Mass Spectrometer equipped with electrospray ionization (ESI) (Thermo Fischer Scientific, Waltham, MA, USA) was used for analysis. The UHPLC separations were performed on a Kinetex XB-C18 column (50 × 3.0 mm; 1.7 μm) (Phenomenex, Torrance, CA, USA). Mobile phases: 0.1% (*v*/*v*) formic acid (A) and 8:2 ACN:0.1% (*v*/*v*) formic acid (B). Separation: 0 min: 10% B, 4 min: 20% B (linear gradient), then isocratic (20% B) from 4 to 8 min followed by a linear gradient to 70% B over 5 min; flow rate: 0.3 mL/min; column temperature: 25 °C; injected volume: 1.0–5 μL. DAD spectra were recorded between 250 and 600 nm. MS parameters: negative ionization mode; parameters were optimized automatically using the built-in software as follows: spray voltage, 2500 V (−); capillary temperature 320 °C; sheath, auxiliary, and spare gases (N2): 47.52, 11.25, and 2.25 arbitrary units, respectively. Full-scan resolution: 70,000 in the scanning range of *m/z* 100–1000. MS/MS scan resolution: 35,000 in the scanning range of *m/z* 80–1000 *m/z*; collision energies: 20, 25, 30, 35, and 45 eV. 

To quantify compounds, extracted ion chromatograms (EICs) of the molecular ions were recovered from the total ion current chromatograms, and an external standard method was applied using EICs. Linear regression analyses of the isolated compounds (PM, IsoPM, FA, FI, and FH) and the standard flavonoid glycoside rutin were performed in the range of 0.2–10.0 ng of their injected amounts, resulting in appropriate r^2^ values (higher than 0.9997 for each compound). Additionally, the quantification of AO and IsoAO was performed according to their previous calibrations, as determined in our recent work [1].

#### 3.4.2. Preparative HPLC 

A Pharmacia LKB HPLC (Uppsala, Sweden) system (2248 pumps, VWM 2141 UV detector) was connected to a preparative HPLC column: Gemini NX-C18 (5 μm), 25 × 1 cm (Phenomenex, Torrance, CA, USA). The eluents were the same as described above. Separation: isocratic, 20% B; flow rate: 3.0 mL/min; injected amount: 300 μL.

#### 3.4.3. Nuclear Magnetic Resonance (NMR) Spectroscopy 

NMR spectra of the isolated compounds were recorded in methanol-*d*_4_ or DMSO-*d*_6_ at 25 °C on a Varian DDR spectrometer (599.9 MHz for 1H and 150.9 MHz for 13C) equipped with a dual 5 mm inverse detection gradient (IDPFG) probe head. Standard pulse sequences and parameters were used to obtain 1D 1H, and various 2D COSY, [1H-13C] HSQC, and [1H-13C] HMBC spectra. Chemical shifts were referenced relative to the solvent resonances.

### 3.5. Computational Method 

All computations were carried out with the Gaussian16 program package (Gaussian Inc., Wallingford, CT, USA, 2016) [49] as described in our previous publication [1].

### 3.6. In Vitro Activity of Compounds on Vero E6 Cell Cultures

The cytotoxic effect of PhEGs isolated from *Forsythia* and *Plantago* tissues was measured on Vero E6 cells (non-human primate origin; *Cercopithecus aethiops*, kidney, epithelial cells; ATCC No. CRL-1586) [52] obtained from the National Biosafety Laboratory, National Public Health Institute, Hungary [53]. The cells were maintained in a DMEM medium (Lonza) containing 10% FBS (Gibco, Thermo Fisher Scientific, Waltham, MA, USA) and supplemented with 2 mM of L-glutamine (Lonza), 1% non-essential amino acids (Gibco), 1 mM sodium pyruvate (Sigma-Aldrich, St. Louis, MO, USA), 1% penicillin–streptomycin (10,000 units penicillin and 10 mg streptomycin/mL, Gibco), and seeded into 96-well plates (5500 cells per well in 100 μL of medium).

Stock solutions of the compounds (20 mM) were prepared with DMSO and diluted with serum-free DMEM, resulting in a final concentration of 0.04–100 μM. The cells were incubated with the compounds, while the control cells were treated with a serum-free medium or serum-free medium containing DMSO (0.5% *v*/*v*) at 37 °C in a humidified atmosphere containing 5% CO_2_. A well-known antiviral agent, chloroquine diphosphate salt, was used at the same concentration range as a positive control. After a 24 or 48 h incubation period, the cells were washed with a serum-free medium (three times, centrifugation: 1000 rpm, 5 min); then, cell viability was determined by alamarBlue assay. For this, 20 μL alamarBlue (Resazurin sodium salt, Sigma-Aldrich, St. Louis, MO, USA) solutions (0.15 mg/mL, dissolved in PBS, pH = 7.4) were added to each well. Following a 4 h incubation period, the fluorescence was measured using a Synergy H4 multi-mode microplate reader (BioTek, Winooski, VT, USA) (λex = 530/30 and at λem = 610/10 nm). 

All measurements were performed in quadruplicates, and the mean cytotoxicity percentage values, together with the standard error of the mean (SEM), were represented on the graphs.

## 4. Conclusions

The PhEG composition of underground organs of *P. lanceolata*, *P. major*, and *P. media*, the leaves of *F. suspensa*, *F. europaea*, and *F. × intermedia*, as well as the fruit wall and seed parts of *F. suspensa* and *F. europaea* fruits collected at different ripening stages were analyzed. The comparative analysis confirmed a tissue-specific accumulation and decomposition of selected PhEGs, resulting in the determination of optimum sources for their isolation. Optimized heat treatment of *P. major* rhizomes and *F. suspensa* unripe fruit walls containing extraordinarily high amounts of PM and FA, respectively, resulted in the isomerization of these PhEGs, also allowing the isolation of their conversion products, IsoPM, FH, and FI, in the highest yield reported so far in the plant kingdom. To confirm their structures, NMR analyses of isolated PhEGs and molecular modeling interpreting the isomerization processes in silico were conducted. The UHPLC-HR-MS/MS study of the closely related isomers also confirmed their on-line discrimination, since baseline separation could be achieved for all isomers and characteristic differences could be observed between some key ion intensities in their MS/MS spectra. The PhEGs, PM, IsoPM, FA, FH, and FI, isolated from their optimum sources, showed no cytotoxic activity on non-human primate Vero E6 cells, supporting the safe use of these compounds as natural medicines and allowing to test their antiviral potency against the SARS-CoV-2 virus. 

## Figures and Tables

**Table 1:** High-resolution mass-spectral (negative ion mode) data for compounds detected in *Plantago major* rhizome (**P1**–**P4**) and *Forsythia suspensa* fruit wall (**S1**–**S6**) extracts.

Ret. Time ^a^	Plant Sample		Formula	Detected Formula	Detected Ion	Calculated *m*/*z*	Found *m*/*z*	Diff (ppm)
	*P. major* ^b^	*F. suspensa* ^c^						
	Compounds							
7.02		**S1**	C_29_H_36_O_15_	[M-H]^−^	C_29_H_35_O_15_	623.19705	623.19733	0.455
7.49	**P1**		C_29_H_36_O_16_	[M-H]^−^	C_29_H_35_O_16_	639.19196	639.19336	2.188
7.89		**S2**	C_27_H_30_O_16_	[M-H]^−^	C_27_H_29_O_16_	609.14501	609.14557	0.918
8.04		**S3**	C_29_H_36_O_15_	[M-H]^−^	C_29_H_35_O_15_	623.19705	623.19739	0.551
8.38		**S4**	C_29_H_36_O_15_	[M-H]^−^	C_29_H_35_O_15_	623.19705	623.19745	0.647
8.94		**S5**	C_29_H_36_O_15_	[M-H]^−^	C_29_H_35_O_15_	623.19705	623.19757	0.840
8.98	**P2**						623.19849	2.316
9.75	**P3**		C_29_H_36_O_16_	[M-H]^−^	C_29_H_35_O_16_	639.19196	639.19324	2.001
10.46		**S6**	C_29_H_36_O_15_	[M-H]^−^	C_29_H_35_O_15_	623.19705	623.19763	0.936
10.44	**P4**						623.19812	1.722

^a^ HPLC-MS retention times (min) and names of compounds (P1–P4, S1–S6) correspond to those in Figure 2 and Figure 3. ^b^ Data refer to the *P. major* rhizome sample marked with collection number 2. ^c^ Data refer to the *F. suspensa* unripe fruit wall sample marked with collection number 1.

**Table 2:** Relative intensities of selective fragment ions, generated from the molecular ions of isolated phenylethanoid glycosides (PhEGs) by collision-induced dissociation (CID ^a^), followed by HPLC-HR-MS/MS analyses of three amounts of injected PhEGs.

PhEGs ^b^	Injected Amounts, ng	Selective Fragment Ions (SFIs), *m/z*						
		161	179	315	461	477	623	639
		Relative Intensities (%) ^c^						
PM	1	54.9	1.54	0.72	-	17.6	-	12.5
	5	56.7	1.64	0.97	-	18.4	-	12.7
	10	57.2	1.53	1.10	-	17.8	-	13.3
		56.3	1.57	0.93	-	17.9	-	12.8
IsoPM	1	31.0	0.31	0.65	-	15.1	-	33.2
	5	35.0	0.78	0.66	-	15.0	-	34.1
	10	34.7	0.90	0.71	-	15.6	-	33.8
		33.6	0.66	0.67	-	15.2	-	33.7
FA	1	53.6	10.4	-	8.22	-	15.0	-
	5	52.8	10.8	-	8.45	-	14.6	-
	10	57.7	10.0	-	8.20	-	16.4	-
		54.7	10.4	-	8.29	-	15.3	-
FI	1	41.7	11.8	-	7.10	-	15.8	-
	5	43.5	12.1	-	6.24	-	15.7	-
	10	45.6	13.3	-	6.73	-	17.2	-
		43.6	12.4	-	6.69	-	16.2	-
FH	1	48.9	8.71	-	8.51		18.7	-
	5	50.3	8.67	-	8.57	-	19.2	-
	10	48.8	8.54	-	8.00	-	17.8	-
		49.3	8.64	-	8.36	-	18.6	-
AO	1	72.7	2.20	1.15	11.6	-	6.22	
	5	68.7	1.96	1.17	11.0	-	6.00	-
	10	67.2	1.92	1.20	10.4	-	5.59	-
		69.5	2.03	1.17	11.0	-	5.94	-
IsoAO	1	57.4	2.51	0.89	14.3	-	13.7	-
	5	60.6	2.61	1.00	15.6	-	14.1	-
	10	56.3	2.32	1.03	14.5	-	14.0	-
		58.1	2.48	0.97	14.8	-	13.9	-

Italic printed data are average results, calculated from the relative ion intensities of the three different injected amounts (1, 5, and 10 ng) of PhEGs. ^a^ A CID energy of 30 eV for the fragmentation of plantamajoside (PM) and isoplantamajoside (IsoPM) and that of 35 eV for the fragmentation of FA, FI, FH, AO, and IsoAO, were used. ^b^ PhEGs plantamajoside, isoplantamajoside, forsythoside A, forsythoside I, forsythoside H, acteoside, and isoacteoside are marked with PM, IsoPM, FA, FI, FH, AO, and IsoAO, respectively. ^c^ Intensities are expressed as percentages of the total product ion current (Full MS2).

**Table 3:** Composition of the dried underground organ samples of three *Plantago* species, determined by UHPLC-MS.

Species	Organ Sample ^a^	Coll. No ^b^	Amounts of Compounds in the Dried Samples (mg/g) ^c^			
			PM	IsoPM	AO	IsoAO
*Plantago major*	root, young plants	1	60.5	3.64	7.34	1.24
		2	37.5	0.48	6.26	0.38
	rhizome, several-year-old plants	1	80.6	2.17	1.56	0.99
		2	82.8	2.09	6.00	0.58
		3	66.3	1.25	7.64	0.53
	root, several-year-old plants	1	20.2	3.24	14.1	1.97
		2	16.6	2.02	10.1	2.11
		3	18.0	0.29	10.4	0.55
*Plantago lanceolata*	root, young plants	1	7.75	0.91	29.2	1.13
		2	10.8	0.87	43.0	2.20
		3	8.35	0.25	38.0	2.30
	rhizome, several-year-old plants	1	15.1	1.68	30.3	3.73
		2	11.6	0.99	28.6	2.40
		3	8.09	0.13	13.9	0.52
	root, several-year-old plants	1	11.4	1.59	16.3	4.59
		2	6.98	0.79	9.45	2.21
		3	10.4	0.25	19.3	2.49
*Plantago media*	root, young plants	1	7.99	2.73	83.9	14.1
		2	10.7	3.56	73.5	10.1
	rhizome, several-year-old plants	1	17.0	3.56	86.8	16.1
		2	9.90	1.65	99.7	21.9
		3	5.22	2.07	53.0	18.1
	root, several-year-old plants	1	18.7	5.79	86.5	33.0
		2	18.8	6.76	86.7	30.5
		3	5.83	4.54	54.7	30.4

^a^ Root system of young plants (which do not develop rhizomes) and separately collected roots and rhizomes of several-year-old plants were analyzed. ^b^ Samples marked with collection numbers 1–3 were harvested from different localities of *Plantago* plants. ^c^ Values are the averages of three separate extractions. Differences could be characterized by the relative standard deviation (RSD) values, ranging from 3.2% (PM in *P. major* root of young plant, Coll. No 2) to 6.8% (IsoAO in *P. major* rhizome of several year-old plant, Coll. No 2). PM: plantamajoside, IsoPM: isoplantamajoside, AO: acteoside, IsoAO: isoacteoside.

**Table 4:** Composition of the dried seed and fruit wall parts of *F. suspensa* and *F. europaea* fruits harvested at different maturity stages (StA, StB, and StC), determined by UHPLC-MS.

Species	Tissue ^a^	Maturity Stage ^b^	Coll. No ^c^	Amounts of Compounds in the Dried Tissues (mg/g) ^d^					
				FA	FI	FH	AO	IsoAO	Rutin
*Forsythia suspensa*	Fruit wall	StA	1	87.0	5.93	0.41	2.72	3.33	0.82
			2	73.8	5.90	0.30	1.69	2.06	0.52
		StB	1	20.6	1.25	-	0.17	1.18	0.14
		StC	1	1.44	-	-	-	0.15	0.11
			2	1.88	-	-	-	0.062	0.023
	Seed	StA	1	58.2	3.68	0.13	0.32	2.00	1.00
			2	59.1	3.04	0.089	0.37	1.33	1.22
		StB	1	53.7	4.15	0.11	0.60	3.69	0.86
		StC	1	59.2	5.15	0.14	0.25	3.95	1.18
			2	64.1	4.03	0.074	-	1.35	1.51
*Forsythia europaea*	Fruit wall	StA	1	-	-	-	77.3	11.6	2.14
			2	-	-	-	65.5	9.2	1.04
		StC	1	-	-	-	2.52	0.62	1.43
			2	-	-	-	2.31	0.58	0.44
	Seed	StA	1	-	-	-	19.3	1.81	4.76
			2	-	-	-	10.4	0.89	4.66
		StC	1	-	-	-	1.97	0.13	2.43
			2	-	-	-	1.13	0.08	0.69

^a^ Fruit wall and seed parts of the fruits were manually separated. ^b^ Maturity stages (StA, StB, StC) correspond to unripe, green, closed fruits (StA), ripe, yellow-brown, closed fruits (StB), and ripe, yellow-brown, opened fruits (StC). ^c^ Collection numbers 1 and 2 correspond to two different individuals of *Forsythia* plants we sampled. ^d^ Values are the averages of three separate extractions. Differences could be characterized by the relative standard deviation (RSD) values, ranging from 2.0% (FA in *F. suspensa* seed at maturity stage StA, Coll. No 1) to 9.3% (IsoAO in *F. suspensa* fruit wall at maturity stage StC, Coll. No 2). FA: forsythoside A, FI: forsythoside I, FH: forsythoside H, AO: acteoside, IsoAO: isoacteoside.

## Data Availability

The data presented in this study are available on request from the corresponding author. The data are not publicly available due to privacy.

## Supplementary Materials

The following are available online at https://www.mdpi.com/article/10.3390/ijms22083880/s1, Table S1. Composition of the dried leaf samples of *Forsythia × intermedia* (represented by six cultivars), *F. europaea* and *F. suspensa*, determined by UHPLC-MS, Table S2. Computed enthalpies (∆*H*), Gibbs free energies (∆*G*), entropies (∆*S*) and carbonylicity percentage (CA%) of the isomers of FA, FI and FH, Table S3. Computed enthalpies (∆*H*) of structures A, B, C and D and activation enthalpies (∆*H‡*) of transition stages TS1 and TS2 in the isomerisation of PM and FA, Figure S1. UHPLC-UV (λ = 330 nm) chromatogram of isolated plantamajoside (A), isoplantamajoside (B), forsythoside I (C), forsythoside H (D), forsythoside A (E) and that of the mixture of these compounds (F), Figure S2. The acyl transfer reaction of PM producing IsoPM. The enthalpy (∆*H*, in kJ mol^–1^), Gibbs free energy (∆*G*, in kJ mol^–1^), entropy (∆*S*, in J mol^–1^ K^–1^) and carbonylicity percentage (CA%) values are illustrated, Figure S3. The acyl transfer reaction of FA producing FI and FH. The corresponding enthalpy, Gibbs free energy, entropy and carbonylicity percentage values are shown in Table S2, Figure S4. The generalized reaction mechanism of the acyl transfer isomerization of PM, determined by computational method using an explicit-implicit solvent model with 7 water molecules. Computed enthalpies of structures A, B, C and D and activation enthalpies of transition stages TS1 and TS2 can be found in the Table S3.

## References

[1] Zürn M., Tóth G., Kraszni M., Sólyomváry A., Mucsi Z., Deme R., Rózsa B., Fodor B., Molnár-Perl I., Horváti K. (2019). Galls of European Fraxinus trees as new and abundant sources of valuable phenylethanoid and coumarin glycosides. Ind. Crop. Prod..

[2] Gonçalves S., Romano A. (2016). The medicinal potential of plants from the genus Plantago (Plantaginaceae). Ind. Crop. Prod..

[3] Lukova P., Dimitrova-Dyulgerova I., Karcheva-Bahchevanska D., Mladenov R., Iliev I., Nikolova M. (2017). Comparative morphological and qualitative phytochemical analysis of Plantago media L. leaves with P. major L. and P. lanceolata L. leaves. Int. J. Med. Res. Pharm. Sci..

[4] Grigore A., Bubueanu C., Pirvu L., Ionita L., Toba G. (2015). Plantago lanceolata L. crops—Source of valuable raw material for various industrial applications. Sci. Pap. Ser. A Agron..

[5] Samuelsen A.B. (2000). The traditional uses, chemical constituents and biological activities of Plantago major L. A review. J. Ethnopharmacol..

[6] Souza C., Barreto R., Soares D. (2008). First report of downy mildew on Plantago major caused by Peronospora alta in Brazil. Australas. Plant Dis. Notes.

[7] Ha Y.-H., Kim C., Choi K., Kim J.-H. (2018). Molecular phylogeny and dating of Forsythieae (Oleaceae) provide insight into the Miocene history of Eurasian temperate shrubs. Front. Plant Sci..

[8] Deyama T., Kobayashi H., Nishibe S., Tu P., Attaur R. (2006). Isolation, Structure Elucidation and Bioactivities of Phenylethanoid Glycosides from Cistanche, Forsythia and Plantago Plants. Studies in Natural Products Chemistry.

[9] Nishibe S. (2002). The plant origins of herbal medicines and their quality evaluation. Yakugaku Zasshi.

[10] Alipieva K., Korkina L., Orhan I.E., Georgiev M.I. (2014). Verbascoside—A review of its occurrence, (bio)synthesis and pharmacological significance. Biotechnol. Adv..

[11] Noro Y., Hisata Y., Okuda K., Kawamura T., Tanaka T., Nishibe S. (1992). Phenylethanoid Glycoside in the Leaves of Forsythia spp.. Shoyakugaku Zasshi.

[12] Wang F.N., Ma Z.Q., Liu Y., Guo Y.Z., Gu Z.W. (2009). New Phenylethanoid Glycosides from the Fruits of Forsythia Suspense (Thunb.) Vahl. Molecules.

[13] Wang Z., Xia Q., Liu X., Liu W., Huang W., Mei X., Luo J., Shan M., Lin R., Zou D. (2018). Phytochemistry, pharmacology, quality control and future research of Forsythia suspensa (Thunb.) Vahl: A review. J. Ethnopharmacol..

[14] Tóth G., Sólyomváry A., Boldizsár I., Noszál B. (2014). Characterization of enzyme-catalysed endogenous β-hydroxylation of phenylethanoid glycosides in Euphrasia rostkoviana Hayne at the molecular level. Process Biochem..

[15] Tóth G., Barabás C., Tóth A., Kéry Á., Béni S., Boldizsár I., Varga E., Noszál B. (2016). Characterization of antioxidant phenolics in Syringa vulgaris L. flowers and fruits by HPLC-DAD-ESI-MS. Biomed. Chromatogr..

[16] Tóth G., Alberti Á., Sólyomváry A., Barabás C., Boldizsár I., Noszál B. (2015). Phenolic profiling of various olive bark-types and leaves: HPLC–ESI/MS study. Ind. Crop. Prod..

[17] Ravn H.W., Mondolot L., Kelly M.T., Lykke A.M. (2015). Plantamajoside—A current review. Phytochem. Lett..

[18] Fons F., Tousch D., Rapior S., Gueiffier A., Roussel J.L., Gargadennec A., Andary C. (1999). Phenolic profiles of untransformed and hairy root cultures of Plantago lanceolata. Plant Physiol. Biochem..

[19] Fons F., Gargadennec A., Gueiffier A., Roussel J.L., Andary C. (1998). Effects of cinnamic acid on polyphenol production in Plantago lanceolata. Phytochemistry.

[20] Budzianowska A., Kikowska M., Małkiewicz M., Karolak I., Budzianowski J. (2019). Phenylethanoid glycosides in Plantago media L. organs obtained in in vitro cultures. Acta Biol. Cracov. Bot..

[21] Miyase T., Ishino M., Akahori C., Ueno A., Ohkawa Y., Tanizawa H. (1991). Phenylethanoid glycosides from Plantago asiatica. Phytochemistry.

[22] Jiao J., Gai Q.-Y., Luo M., Wang W., Gu C.-B., Zhao C.-J., Zu Y.-G., Wei F.-Y., Fu Y.-J. (2013). Comparison of main bioactive compounds in tea infusions with different seasonal Forsythia suspensa leaves by liquid chromatography–tandem mass spectrometry and evaluation of antioxidant activity. Food Res. Int..

[23] Qu H., Li B., Li X., Tu G., Lü J., Sun W. (2008). Qualitative and quantitative analyses of three bioactive compounds in different parts of Forsythia suspensa by high-performance liquid chromatography-electrospray ionization-mass spectrometry. Microchem. J..

[24] Jia J., Zhang F., Li Z., Qin X., Zhang L. (2015). Comparison of fruits of Forsythia suspensa at two different maturation stages by NMR-based metabolomics. Molecules.

[25] Kitagawa S., Hisada S., Nishibe S. (1984). Phenolic compounds from Forsythia leaves. Phytochemistry.

[26] Damtoft S., Franzyk H., Rosendal Jensen S. (1994). Biosynthesis of iridoids in Forsythia spp.. Phytochemistry.

[27] Li C., Dai Y., Duan Y.-H., Liu M.-L., Yao X.-S. (2014). A new lignan glycoside from Forsythia suspensa. Chin. J. Nat. Med..

[28] Guo H., Liu A.-H., Li L., Guo D.-A. (2007). Simultaneous determination of 12 major constituents in Forsythia suspensa by high performance liquid chromatography—DAD method. J. Pharmaceut. Biomed..

[29] Qi M., Zhao S., Zhou B., Zhang M., Zhang H., Wang Y., Hu P. (2019). Probing the degradation mechanism of forsythiaside A and simultaneous determination of three forsythiasides in Forsythia preparations by a single marker. J. Sep. Sci..

[30] Kuo P.-C., Hung H.-Y., Nian C.-W., Hwang T.-L., Cheng J.-C., Kuo D.-H., Lee E.-J., Tai S.-H., Wu T.-S. (2017). Chemical Constituents and Anti-inflammatory Principles from the Fruits of Forsythia suspensa. J. Nat. Prod..

[31] Kuang H.-X., Xia Y.-G., Liang J., Yang B.-Y., Wang Q.-H. (2011). Lianqiaoxinoside B, a Novel Caffeoyl Phenylethanoid Glycoside from Forsythia suspensa. Molecules.

[32] Qu H., Zhang Y., Chai X., Sun W. (2012). Isoforsythiaside, an antioxidant and antibacterial phenylethanoid glycoside isolated from Forsythia suspensa. Bioorg. Chem..

[33] Ahn J.H., Jo Y.H., Kim S.B., Turk A., Oh K.-E., Hwang B.Y., Lee K.Y., Lee M.K. (2018). Identification of antioxidant constituents of the aerial part of Plantago asiatica using LC–MS/MS coupled DPPH assay. Phytochem. Lett..

[34] Geng F., Yang L., Chou G., Wang Z. (2010). Bioguided isolation of angiotensin-converting enzyme inhibitors from the seeds of Plantago asiatica L.. Phytother. Res..

[35] Kim D.-S., Woo E.-R., Chae S.-W., Ha K.-C., Lee G.-H., Hong S.-T., Kwon D.-Y., Kim M.-S., Jung Y.-K., Kim H.-M. (2007). Plantainoside D protects adriamycin-induced apoptosis in H9c2 cardiac muscle cells via the inhibition of ROS generation and NF-κB activation. Life Sci..

[36] Law A.H.-Y., Yang C.L.-H., Lau A.S.-Y., Chan G.C.-F. (2017). Antiviral effect of forsythoside A from Forsythia suspensa (Thunb.) Vahl fruit against influenza A virus through reduction of viral M1 protein. J. Ethnopharmacol..

[37] Li H., Wu J., Zhang Z., Ma Y., Liao F., Zhang Y., Wu G. (2011). Forsythoside A inhibits the avian infectious bronchitis virus in cell culture. Phytother. Res..

[38] Kernan M.R., Amarquaye A., Chen J.L., Chan J., Sesin D.F., Parkinson N., Ye Z., Barrett M., Bales C., Stoddart C.A. (1998). Antiviral phenylpropanoid glycosides from the medicinal plant Markhamia lutea. J. Nat. Prod..

[39] Brandão G.C., Kroon E.G., Souza D.E.R., Souza Filho J.D., Oliveira A.B. (2013). Chemistry and Antiviral Activity of Arrabidaea pulchra (Bignoniaceae). Molecules.

[40] Ravn H., Brimer L. (1988). Structure and antibacterial activity of plantamajoside, a caffeic acid sugar ester from Plantago major subsp. major. Phytochemistry.

[41] Li C., Liu Y., Abdulla R., Aisa H.A., Suo Y. (2014). Characterization and identification of chemical components in Neopicrorhiza scrphulariiflora roots by liquid chromatography-electrospray ionization quadrupole time-of-flight tandem mass spectrometry. Anal. Methods.

[42] Michalak B., Filipek A., Chomicki P., Pyza M., Woźniak M., Żyżyńska-Granica B., Piwowarski J.P., Kicel A., Olszewska M.A., Kiss A.K. (2018). Lignans from Forsythia x Intermedia leaves and flowers attenuate the pro-inflammatory function of leukocytes and their interaction with endothelial cells. Front. Pharmacol..

[43] Kitagawa S., Nishibe S., Benecke R., Thieme H. (1988). Phenolic Compounds from Forsythia Leaves. II. Chem. Pharm. Bull..

[44] Mucsi Z., Chass G.A., Csizmadia I.G. (2008). Amidicity change as a significant driving force and thermodynamic selection rule of transamidation reactions. A synergy between experiment and theory. J. Phys. Chem. B.

[45] Mucsi Z., Chass G.A., Viskolcz B., Csizmadia I.G. (2008). Quantitative scale for the extent of conjugation of carbonyl groups: “carbonylicity” percentage as a chemical driving force. J. Phys. Chem. A.

[46] Kovács E., Rózsa B., Csomos A., Csizmadia I.G., Mucsi Z. (2018). Amide Activation in Ground and Excited States. Molecules.

[47] Becke A.D. (1993). Density-functional thermochemistry. III. The role of exact exchange. J. Chem. Phys..

[48] Tomasi J., Mennucci B., Cammi R. (2005). Quantum mechanical continuum solvation models. Chem. Rev..

[49] Frisch M.J., Trucks G.W., Schlegel H.B., Scuseria G.E., Robb M.A., Cheeseman J.R., Scalmani G., Barone V., Petersson G.A., Nakatsuji H. (2016). Gaussian 16 Revision B.01.

[50] Mucsi Z., Szabó A., Hermecz I., Kucsman A., Csizmadia I.G. (2005). Modeling rate-controlling solvent effects. The pericyclic meisenheimer rearrangement of N-propargylmorpholine N-oxide. J. Am. Chem. Soc..

[51] Pei S., Yang X., Wang H., Zhang H., Zhou B., Zhang D., Lin D. (2015). Plantamajoside, a potential anti-tumor herbal medicine inhibits breast cancer growth and pulmonary metastasis by decreasing the activity of matrix metalloproteinase-9 and -2. BMC Cancer.

[52] Emeny J.M., Morgan M.J. (1979). Regulation of the interferon system: Evidence that Vero cells have a genetic defect in interferon production. J. Gen. Virol..

[53] Palyi B., Magyar N., Henczko J., Szalai B., Farkas A., Strecker T., Takacs M., Kis Z. (2018). Determining the effect of different environmental conditions on Ebola virus viability in clinically relevant specimens. Emerg. Microbes Infect..

