# Supplementary material for: Tissue-Specific Accumulation and Isomerization of Valuable Phenylethanoid Glycosides from Plantago and Forsythia Plants
Source: Int J Mol Sci. 2021 Apr 9;22(8):3880. doi: 10.3390/ijms22083880 (PMC8069251; doi:10.3390/ijms22083880)
Supplement: Supplementary file 1 [file ijms-22-03880-s001.pdf]

Supplementary Material

The supplementary material shows the NMR data of isolated PhEGs, the UHPLC-UV chromatograms of these PhEGs (Figure S1), the composition of the leaf samples of *Forsythia* × *intermedia*, *F. europaea* and *F. suspensa* (Table S1), and the computational modeling results of the plantamajoside and forsythoside A isomerization (Tables S2, S3, Figures S2–S4).

The NMR data of isolated PhEGs

Forsythoside A (FA):

^{1}H-NMR (DMSO-d_6): 7.50 d (*J*=15.6 Hz), 7.07 s, 7.01 d (*J*=8.5 Hz), 6.75 d (J=8.5 Hz), 6.63 d (J=7.8 Hz), 6.62 s, 6.49 d (J= 7.8 Hz), 6.25 d (J=15.6 Hz), 4.66 t (J=9.5 Hz), 4.49 s, 4.30 d (J=7.8 Hz), 3.83 m, 3.61 m, 3.59 m, 3.55 t (J=9Hz), 3.50 d (J=12.8 Hz), 3.44 m, 3.40 dd (J=7.8, 9.0), 3.36 dd (J=8.9, 10.2), 3.33 m, 3.30 dd (J=7.4, 12.8 Hz), 3.11 m, 2.65 m, 1.04 d (6.0);

^{13}C NMR (DMSO-d_6): 165.9, 148.8, 145.6, 145.5 145.0, 143.4, 129.0, 125.5, 121.2, 119.7, 116.3, 115.8, 115.5, 115.0, 113.4, 102.8, 100.5, 74.1, 73.5, 73.1, 72.0, 71.1, 70.7, 70.6, 70.3, 68.5, 66.2, 35.2, 17.9 (data identical as Ref: Wang et al. 2009 [12]).

Forsythoside H (FH):

^{1}H-NMR (DMSO-d_6): 7.49 d (J=16.2 Hz), 7.06 s, 7.02 d (J=7.0 Hz), 6.75 d (J=7.0 Hz), 6.56 d (J=8.2), 6.53 s, 6.41 d (J=8.2 Hz), 6.27 d (J=16.2 Hz), 4.64 t (J=8.5 Hz), 4.60 s, 4.48 d (J=8.4 Hz), 3.84 d (10.2), 3.76 m, 3.64 m, 3.54 m, 3.48 m, 3.45 m, 3.44 m, 3.43 m, 3.41 m, 3.38 m, 3.18 t (J=9.4 Hz), 2.56 m, 1.14 d (J=6.6);

^{13}C NMR (DMSO-d_6): 165.7, 148.8, 145.7, 145.3, 145.0, 143.4, 129.1, 125.6, 121.3, 119.8, 116.1, 115.9, 115.4, 114.9, 114.2, 100.9, 100.2, 75.8, 74.1, 73.4, 72.1, 70.7, 70.5, 70.3, 69.9, 68.5, 66.6, 35.2, 18.0 (data identical as Ref: Wang et al. 2009 [12]).

Forsythoside I (FI):

^{1}H-NMR (DMSO-d_6): 7.47 d (J=15.6 Hz), 7.04 br. s, 7.00 d (J=8.2 Hz), 6.75 d (J=8.2 Hz), 6.63 d (J=8.0), 6.61 s, 6.46 d (J=8.0 Hz), 6.25 d (J=15.6 Hz), 4.89 t (J=8.8 Hz), 4.60 s, 4.35 d (J=7.8 Hz), 3.83 d (9.6), 3.82 m, 3.63 m, 3.62 m, 3.61 m, 3.50 m, 3.48 m, 3.45 m, 3.42 m, 3.30 dd (J=9.0 Hz, 10.2 Hz), 3.20 (J=7.8, 9.0 Hz), 2.69 m, 1.12 d (J=6.6 Hz);

^{13}C NMR (DMSO-d_6): 166.1, 148.4, 145.5, 144.8, 144.6, 143.5, 129.5, 125.6, 121.3, 119.4, 116.3, 115.8, 115.6, 114.9, 114.6, 102.9, 100.5, 77.8, 75.1, 71.9, 71.5, 70.6, 70.4, 70.2, 68.5, 68.1, 66.5, 35.2, 18.0 (data identical as Ref: Wang et al. 2009 [12]).

Plantamajoside (PM):

^{1}H-NMR (CD_3OD): 7.58 d, (J=15.8 Hz), 7.06 d (J=1.8 Hz), 6.97 dd (J=1.9 and 8.2 Hz), 6.78 d (J=8.2 Hz), 6.71 d (J=1.9 Hz), 6.65 d (J=8.0 Hz), 6.55 dd (J=1.9 and 8.0 Hz), 6.30 (d J=15.8 Hz), 4.90 t (J=9.3 Hz), 4.50 d (J=7.5 Hz), 4.39 d (J=7.5 Hz), 4.03 m, 3.90 t (J=9.3 Hz), 3.71 m, 3.66 m, 3.63 m, 3.55 m, 3.52 m, 3.49 m, 3.47 m, 3.30 m, 3.25 t (J=9.0 Hz), 3.17 m, 3.14 m, 2.80 m;

^{13}C-NMR (CD_3OD): 168.8, 150.2, 147.8, 147.1, 146.5, 144.9, 131.9, 128.0, 124.0, 121.4, 117.3, 117.0, 116.8, 115.7, 115.5, 106.2, 104.2, 84.4, 78.4, 78.0, 76.4, 76.2, 75.2, 72.6, 71.5, 71.1, 62.7, 62.5, 37.0 (data identical as Ref: Ravn and Brimer, 1988 [40]).

Isoplantamajoside (IsoPM):

^{1}H-NMR (CD_3OD): 7.60 d, (J=15.9 Hz), 7.10 d (J=1.8 Hz), 6.99 dd (J=1.9 and 8.2 Hz), 6.82 d (J=8.2 Hz), 6.71 d (J=1.9 Hz), 6.67 d (J=8.0 Hz), 6.54 dd (J=1.9 and 8.0 Hz), 6.34 (d J=15.9 Hz), 4.55 m, 4.53 (J=7.5 Hz), 4.45 d (J=7.5 Hz), 4.44 m, 4.05 m, 3.75 m, 3.62 m, 3.58 t (J=9.4 Hz), 3.50 m, 3.43 m, 3.41 t (J=9.4 Hz), 3.35 m, 3.23 t (J=9.0 Hz), 3.19 m, 3.17 m, 2.80 m;

^{13}C-NMR (CD_3OD): 168.9, 150.0, 147.9, 147.2, 146.7, 145.0, 131.8, 128.0, 123.5, 121.7, 117.5, 116.9, 116.7, 115.7, 115.5. 105.9, 104.5, 85.8, 78.5, 77.9, 76.5, 75.9, 75.2, 72.5, 71.5, 70.8, 63.5, 62.7, 37.0 (data identical as Ref: Miyase et al., 1991 [21]).

Table S1

Composition of the dried leaf samples of *Forsythia* × *intermedia* (represented by six cultivars), *F. europaea* and *F. suspensa*, determined by UHPLC-MS.

Species		Coll. No[a]	Amounts of compounds in the dried leaf (mg/g)[b]				
			FA	FI	AO	IsoAO	rutin
Forsythia × *intermedia*	'Lynwood'	1	25.6	0.11	10.1	0.25	3.8
		2	35.9	0.26	17.1	0.56	5.1
	'Melisa'	1	43.7	0.014	28.4	1.38	5.9
		2	12.0	0.008	5.90	0.19	2.1
	'Minigold'	1	45.1	0.013	19.8	0.56	11.4
		2	6.50	0.004	2.75	0.06	1.25
	'Primulina'	1	53.6	0.22	22.2	0.63	8.13
		2	66.4	0.28	24.6	0.50	8.44
	'Spectabilis'	1	18.3	0.007	9.69	0.25	3.19
		2	13.8	0.006	8.19	0.19	1.38
	'Week End'	1	12.0	0.004	4.50	0.06	2.19
		2	36.1	0.013	16.3	0.44	0.31
Forsythia europaea		1	-	-	73.9	7.55	7.28
		2	-	-	73.2	5.81	4.17
Forsythia suspensa		1	96.0	0.60	-	4.15	5.69
		2	80.6	0.42	-	3.03	4.75

[a] Collection numbers 1 and 2 corresponds to two different individuals of *Forsythia* plants we sampled.

[b] Values are the averages of three separate extractions. Differences could be characterized by the relative standard deviation (RSD) values, ranging from 2.9% (FA in *F* × *intermedia* 'Lynwood', collection No 1) to 10.6% (FI in *F* × *intermedia* 'Minigold', collection No 2).

FA: forsythoside A, FI: forsythoside I, FH: forsythoside H, AO: acteoside, IsoAO: isoacteoside.

Table S2

Computed enthalpies (ΔH), Gibbs free energies (ΔG), entropies (ΔS) and carbonylicity percentage (CA%) of the isomers of FA, FI and FH.

isomer	ΔH (kJ mol^{-1})	ΔG (kJ mol^{-1})	ΔS (J mol^{-1} K^{-1})	CA%
FA	5.0	7.3	–7.8	52.9%.
FI	0.0	0.0	0.0	53.0%
FH	3.3	4.9	–5.5	52.5%

Table S3

Computed enthalpies (ΔH) of structures A, B, C and D and activation enthalpies (ΔH‡) of transition stages TS1 and TS2 in the isomerisation of PM and FA.

Isomerizations	Structures and transition stages[a]					
	A	TS1	B	C	TS2	D
PM→IsoPM	0.0	91.3	63.8	18.2	64.7	–15.9
PM→IsoPM-2	0.0	117.6	78.9	26.4	80.6	+25.5
FA→FI	0.0	86.1	66.7	22.4	59[b]	-4.2
FI→FH	-4.2	92.4	64.1	38.8	61[b]	+6.8

[a] Structures (A, B, C, D) and transition stages (TS1, TS2) can be found in Fig. S4.

[b] Estimated values, obtained by scanning along the reaction coordinates.

(± 3–4 kJ mol^{-1})

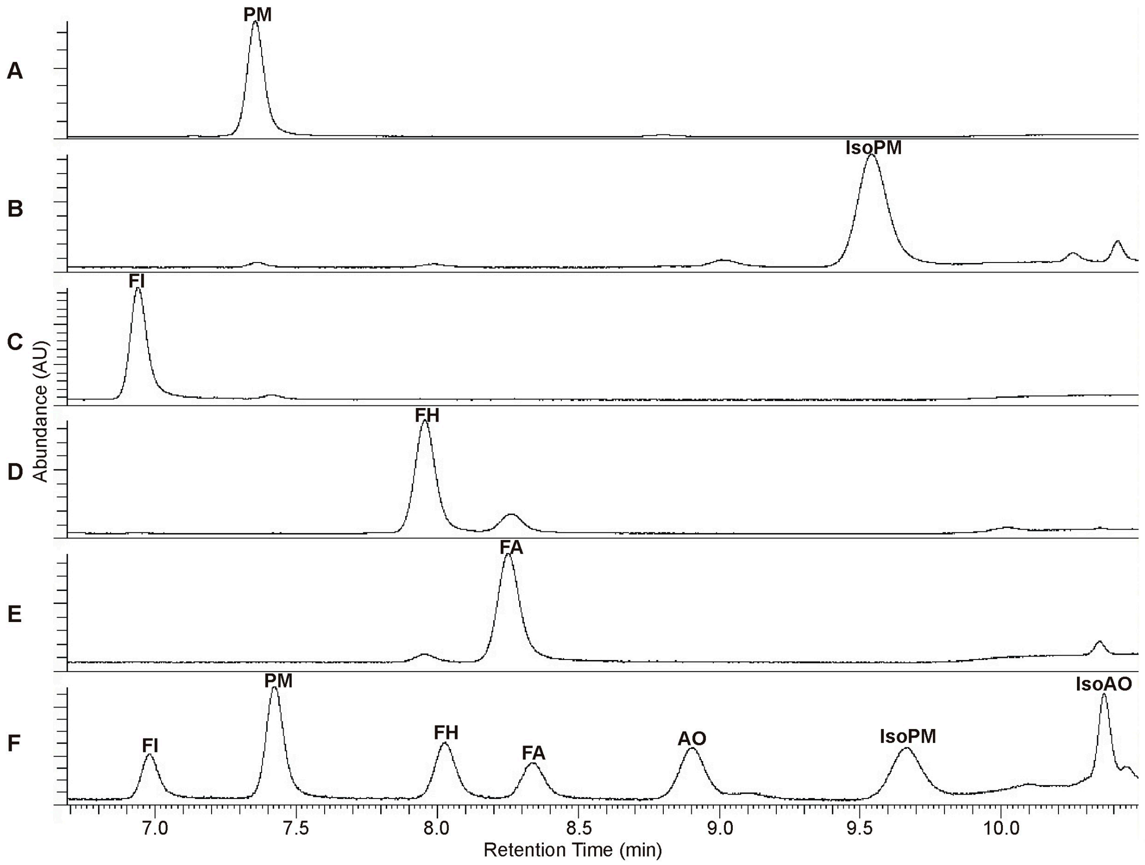

Figure S1.

UHPLC-UV (λ=330 nm) chromatogram of isolated plantamajoside (A), isoplantamajoside (B), forsythoside I (C), forsythoside H (D), forsythoside A (E) and that of the mixture of these compounds (F).

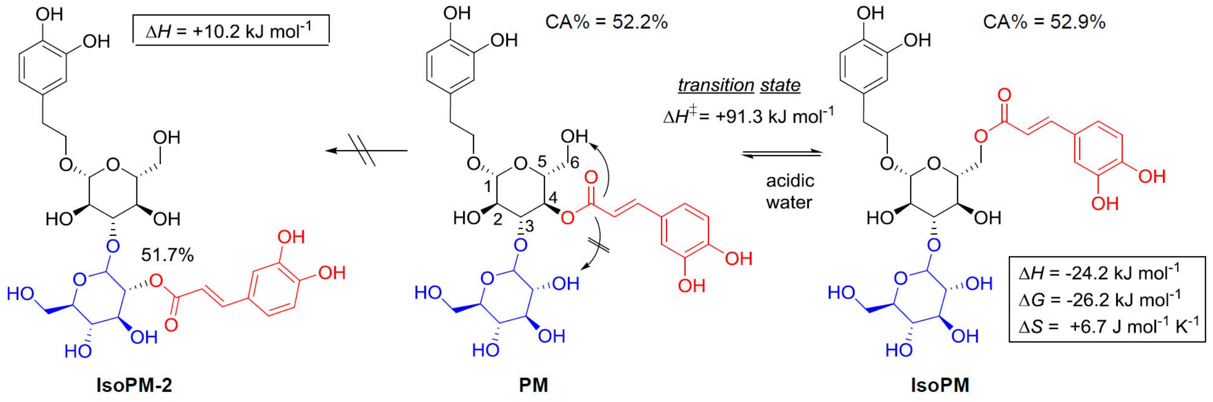

Figure S2.

The acyl transfer reaction of PM producing IsoPM. The enthalpy (ΔH, in kJ mol^{-1}), Gibbs free energy (ΔG, in kJ mol^{-1}), entropy (ΔS, in J mol^{-1} K^{-1}) and carbonylicity percentage (CA%) values are illustrated.

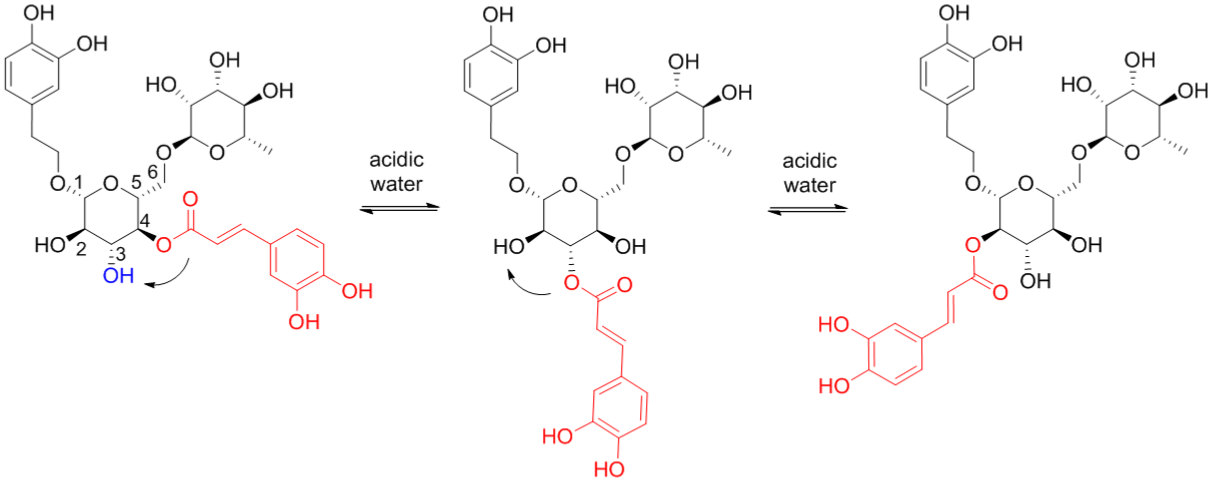

Figure S3.

The acyl transfer reaction of FA producing FI and FH. The corresponding enthalpy, Gibbs free energy, entropy and carbonylicity percentage values are shown in Table S2.

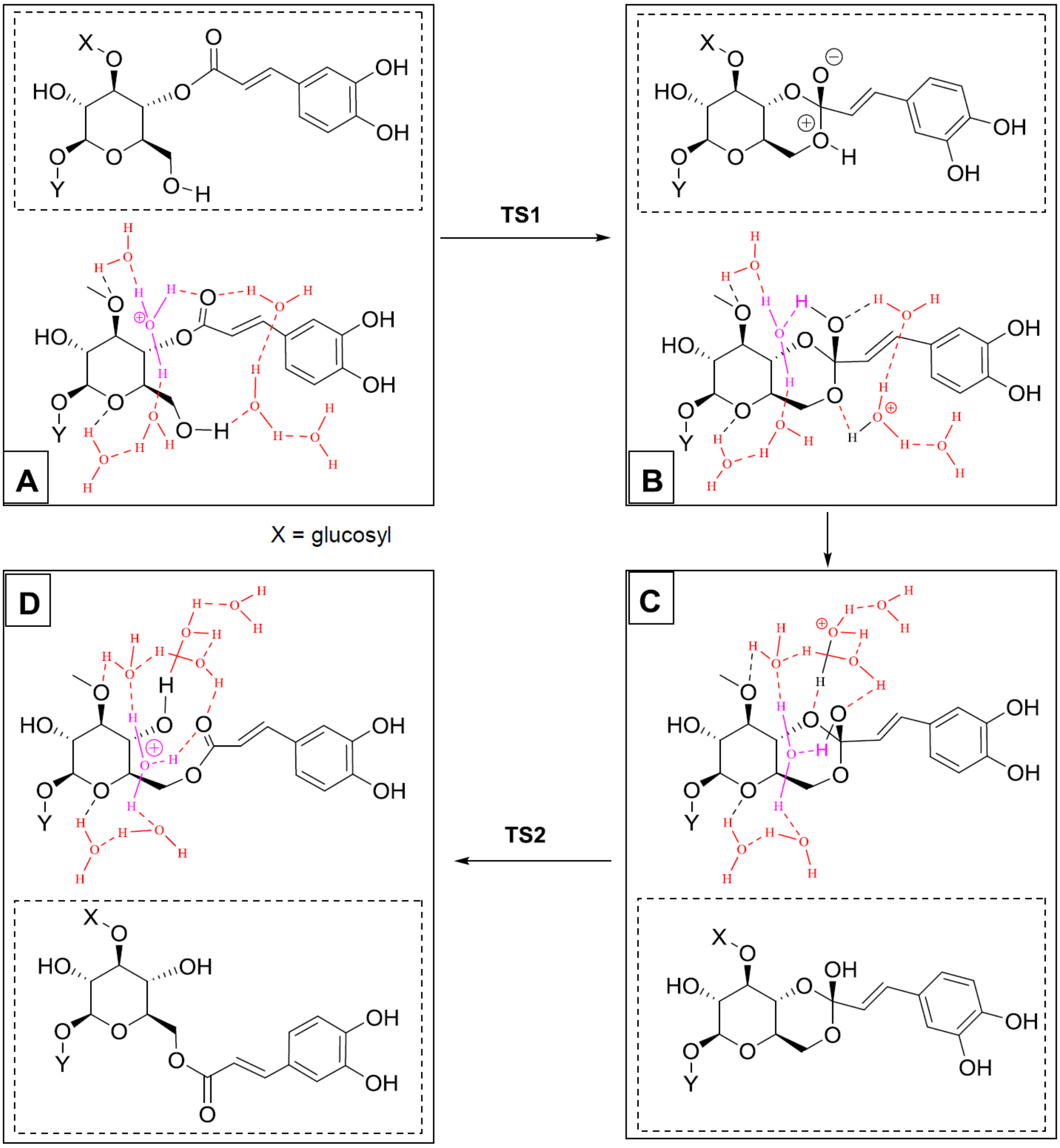

Figure S4.

The generalized reaction mechanism of the acyl transfer isomerization of PM, determined by computational method using an explicit-implicit solvent model with 7 water molecules. Computed enthalpies of structures A, B, C and D and activation enthalpies of transition stages TS1 and TS2 can be found in the Table S3.
